# Frontiers in the Application of RF Vacuum Electronics

**DOI:** 10.1109/ted.2023.3239841

**Published:** 2023-03-03

**Authors:** Carter M. Armstrong, Emma C. Snively, Muhammad Shumail, Christopher Nantista, Zenghai Li, Sami Tantawi, Bill W. Loo, Richard J. Temkin, Robert G. Griffin, Jinjun Feng, Roberto Dionisio, Felix Mentgen, Natanael Ayllon, Mark A. Henderson, Timothy P. Goodman

**Affiliations:** L3Harris Electron Devices, Torrance, CA 90505 USA; SLAC National Accelerator Laboratory, Menlo Park, CA 94025 USA; SLAC National Accelerator Laboratory, Menlo Park, CA 94025 USA; SLAC National Accelerator Laboratory, Menlo Park, CA 94025 USA; SLAC National Accelerator Laboratory, Menlo Park, CA 94025 USA; SLAC National Accelerator Laboratory, Menlo Park, CA 94025 USA; Department of Radiation Oncology and Stanford Cancer Institute, Stanford University School of Medicine, Stanford, CA 94305 USA; Department of Physics and the Plasma Science and Fusion Center, Massachusetts Institute of Technology, Cambridge, MA 02139 USA; Department of Chemistry and the Francis Bitter Magnet Laboratory, Massachusetts Institute of Technology, Cambridge, MA 02139 USA; Beijing Vacuum Electronics Research Institute, Beijing 100015, China; RF Equipment and Technologies Section, European Space Agency (ESA), NL-2200 AG Noordwijk, The Netherlands; RF Equipment and Technologies Section, European Space Agency (ESA), NL-2200 AG Noordwijk, The Netherlands; RF Equipment and Technologies Section, European Space Agency (ESA), NL-2200 AG Noordwijk, The Netherlands; United Kingdom Atomic Energy Authority, Culham Science Centre, OX14 3DB Abingdon, U.K.; Swiss Plasma Center, École Polytechnique Fédérale de Lausanne (EPFL), 1015 Lausanne, Switzerland

**Keywords:** Applications, cancer therapy, dynamic nuclear polarization (DNP), Earth observation, fusion energy, microwave, millimeter-wave (mm-wave), nuclear magnetic resonance (NMR), radio frequency (RF), RF acceleration, satellite communication, terahertz, vacuum electronics, wireless communication

## Abstract

The application of radio frequency (RF) vacuum electronics for the betterment of the human condition began soon after the invention of the first vacuum tubes in the 1920s and has not stopped since. Today, microwave vacuum devices are powering important applications in health treatment, material and biological science, wireless communication—terrestrial and space, Earth environment remote sensing, and the promise of safe, reliable, and inexhaustible energy. This article highlights some of the exciting application frontiers of vacuum electronics.

## I. Introduction

EVERY device scientist and engineer knows full well that while the successful design and prototyping of a new or improved variant is the cause for great personal accomplishment, true satisfaction comes from seeing its use in an application of societal benefit—the answer to the public’s inquiries of “so where is it used, what is it good for?”

Vacuum devices excel in the efficient generation of powerful coherent electromagnetic radiation. As such they found immediate application soon after their invention. The early cavity klystrons and cavity magnetrons developed in the late 1930s/early 1940s, for example, found initial use in radio communication and radar. And the development and application of vacuum electronics devices did not stop there; it continues to this day [1]. In areas such as industrial-scale heating and plasma processing, powering particle accelerators for cancer treatment and exploring the mysteries of nature, national defense and security, and satellite communication and space exploration, radio frequency (RF) vacuum devices continue to play an important role. Indeed, you might be surprised to find that you depend on vacuum electronics technology more than you know. The food you heat in your microwave and your satellite TV and satellite radio reception are made possible with vacuum electronics. Even the historic imagery of Neil Armstrong’s “small step” on the lunar surface in July 1969 depended on a vacuum tube to beam the transmission down to Earth [2]. And since we are talking about space exploration, it is amazing to note that both of the Voyager spacecraft, launched in 1977 and now traveling outside the solar system in interstellar space, are still phoning home with a vacuum electronics-based communication system. And to make sure someone is still here to listen, tubes, specifically klystrons, are used in the Earth asteroid planetary defense system to make sure mankind does not go the way of the dinosaurs [3].

This article highlights examples of advanced and emerging applications of RF vacuum electronics in areas of importance to the advancement of the human condition, including health treatment, material and bio-science, high-speed, high-capacity terrestrial and space communication/information distribution, environmental Earth observation, and the pursuit of means for the commercially viable generation of environmentally safe, reliable, and abundant energy. The topics chosen for this compilation were selected by the first author; print space limitations prevented the inclusion of other worthy candidates. The authors of the individual application summaries are identified in the main body of the text.

This article is focused on the application of vacuum electronics, not on vacuum electronics technology, per se. However, areas for fruitful continued device development are identified in the individual application write-ups. This article is not a vacuum tube technology primer. The reader interested in learning more about vacuum electronics should consult the references, as well as the articles in this Special Issue, along with the many excellent textbooks on RF vacuum device technology [4], [5], [6].

## II. Highlighted Applications

### A. RF Accelerators for FLASH Radiation Therapy^1^

The state-of-the-art in today’s radiation oncology practice is driven by the guiding principles of increasing the therapeutic index, i.e., the balance of tumor eradication versus normal tissue collateral injury and improving the accuracy of dose delivery. Current technology addresses these challenges from multiple fronts, using innovations like multileaf collimators to shape the dose profile. However, one of the most challenging aspects in the practice of radiation therapy (RT) is the dynamic nature of the live human body. Internal organs are moving all the time even during treatment. Hence, techniques such as active imaging to guide motion-adapted RT have been developed. While these techniques improve the treatment quality, it takes a long time to prepare the patient for these protocols and the technology still does not produce the desired treatment precision. One logical solution to this motion problem is to deliver the therapeutic dose precisely to the tumor in a very short period of time so that the body is essentially static during the treatment time [7]. Furthermore, studies have shown that drastically reduced treatment times of a few hundred milliseconds with a dose rate exceeding 40 Gy/s, a so-called FLASH therapy, result in a favorable differential effect between the biological damage accrued in tumors and normal tissue [8], [9]. The preliminary results suggest a revolutionary improvement to the sparing of healthy tissue may be on the horizon.

The treatment techniques available in today’s radiation oncology clinics are dominated by photon irradiation, using *γ*-rays generated from electron beams with energies up to ~15 MeV. To achieve FLASH dose rates using bremsstrahlung-generated *γ*-rays requires that the linear accelerators (linacs) used for that system perform with much higher currents and repetition rates than the state-of-the-art of current medical linacs. While the development of new systems like PHASER [7] may one day be able to reach the high dose rates of FLASH therapy, a significantly more efficient approach is to use the electron beam directly by eliminating the target, or converter, which converts the electron beam energy into bremsstrahlung-generated *γ*-rays. The challenge in this case is that the energy of the electron beam must be increased to the point that the multiple scattering of electrons inside the body does not deliver doses to unintended parts, requiring at least 100 MeV. Direct comparison of simulated treatment plans has shown that very high energy electrons (VHEE) may offer improved dose conformity compared to conventional intensity-modulated RT in many treatment scenarios [10].

Current treatment facilities are limited to beam energies of up to ~20 MeV by the available space, power infrastructure, and linac performance specifications. Achieving a 100-MeV beam is a seemingly trivial task within the accelerator research community, but the length scale of the structure required to get there is typically several meters. The campaign for >100-MeV/m accelerating gradients is a highly active area of research, with a wide range of techniques under study [11], [12]. Beyond the gradient required for practical VHEE therapy, a few key criteria narrow the scope of techniques that could be considered to achieve VHEE beams, chief among these are system reliability, size, cost, and attainable beam charge. Since the RF power required driving the linacs scales quadratically with the operating gradient, to go from the 10 MV/m that is typical in today’s medical linacs to the ~100 MV/m that is needed for VHEE, the RF sources must produce 100 times more peak power. This makes the system large, expensive, and unpractical to use in a clinical setting.

Research conducted at the SLAC National Accelerator Laboratory, Menlo Park, CA, USA, through the D.O.E.’s Accelerator Stewardship Program aims to address these practical obstacles through the development of a high gradient linac and RF pulse compression system that can utilize a compact commercial modulator and klystron producing 6–8 MW at X-band to reach the required VHEE beam energies [13], [14]. The linac design takes advantage of cryogenic cooling and the comparatively high operating frequency, 11.424 GHz, to reach a shunt impedance of over 500 MΩ/m, a metric that measures the ability of the structure to convert the input power into usable accelerating gradient. A key contribution to this high shunt impedance is the optimization of the linac cavities themselves. The structure is designed for a 135° phase advance between cells, achieving the highest possible geometric shunt impedance, with cavity features like re-entrant nose cones determined using a genetic optimization algorithm to maximize the ratio of on-axis accelerating gradient to peak surface field [15].

**Fig. 1.**
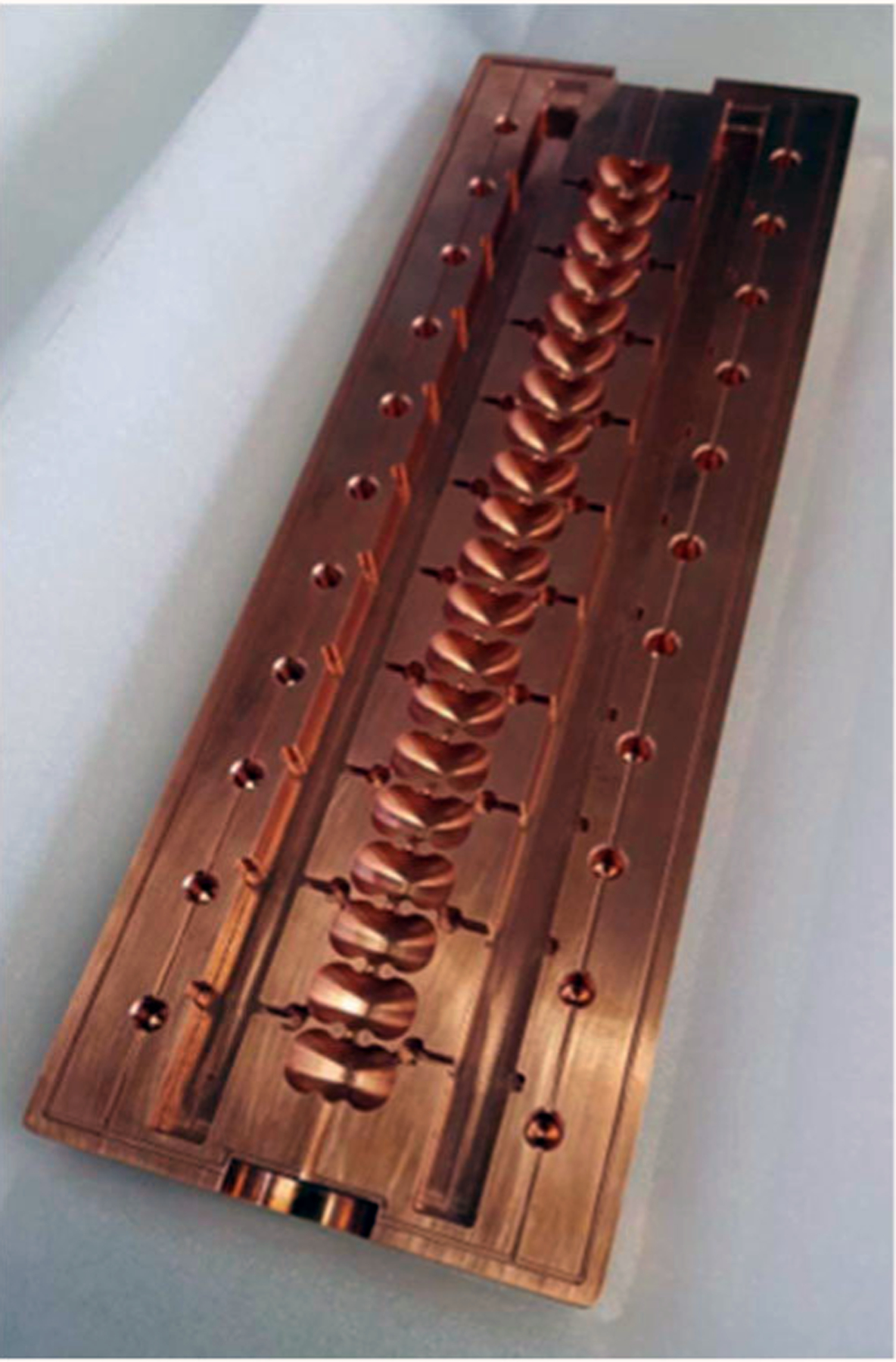
One half of distributed coupling linac designed at SLAC. Each of the 20 cells in this linac is about 13.1 mm long.

The extensive optimization now possible in SLAC’s design process for RF structures relies on advances in computing power and simulation capabilities, like SLAC’s parallel electromagnetic code suite ACE3P, as well as the innovation of distributed power coupling [16]. This method of feeding power to each cell relieves the constraint on the aperture size between cells that is conventionally associated with coupling power along the beam axis and allows more flexibility in the design of the cavity geometry. For previous prototypes developed at SLAC, the accelerator cavities and parallel power distribution manifolds have been assembled out of two oxygen-free copper blocks, with one half of the structure milled out of each side, as shown in Fig. 1. For the VHEE system currently in development, the accelerator will have four parallel manifolds, each feeding every fourth cell to accommodate the 135° phase advance, resulting in a structure that will be assembled out of four blocks.

With the high shunt impedance of this one-meter structure, a 100-MeV/m gradient can be achieved with an input of only 19 MW, putting this peak power into the range that can be achieved through RF pulse compression of the >4-*μ*s pulse from the commercial RF source down to a few hundred nanoseconds. The design of the pulse compressor for the VHEE linac is an extension of the original 1974 compressor concept [17] in which the energy of the long RF pulse is stored up in two cavities with high quality factor *Q*_0_, before a phase flip in the incoming RF initiates the process of discharging the cavities. The new VHEE compressor relies on two polarized modes in a single cavity, like other recent designs used at SLAC [18], but with a corrugated cylindrical cavity that has a high *Q*_0_, around 400 000 at room temperature, while allowing better access for coupling and tuning at the ends of the cylinder. This pulse compression scheme opens new opportunities to harness compact RF sources for high gradient accelerators.

While the design of SLAC’s accelerator is intended to be compatible with a commercially marketed modulator, the reality is the number of companies with products that meet the needs of a clinical VHEE system is very limited. Widespread adoption of a VHEE therapy machine to provide FLASH treatments would be bolstered by increased availability of affordable modulator products. Further RF source development to improve efficiency, increase peak power, and reduce the footprint of these high-frequency systems would be beneficial not only for the deployment of a new generation of medical accelerator technology, but also for industrial and national security applications, in which the available space and power budget may be heavily constrained.

### B. Magnetic Resonance Spectroscopy^2^

**Fig. 2.**
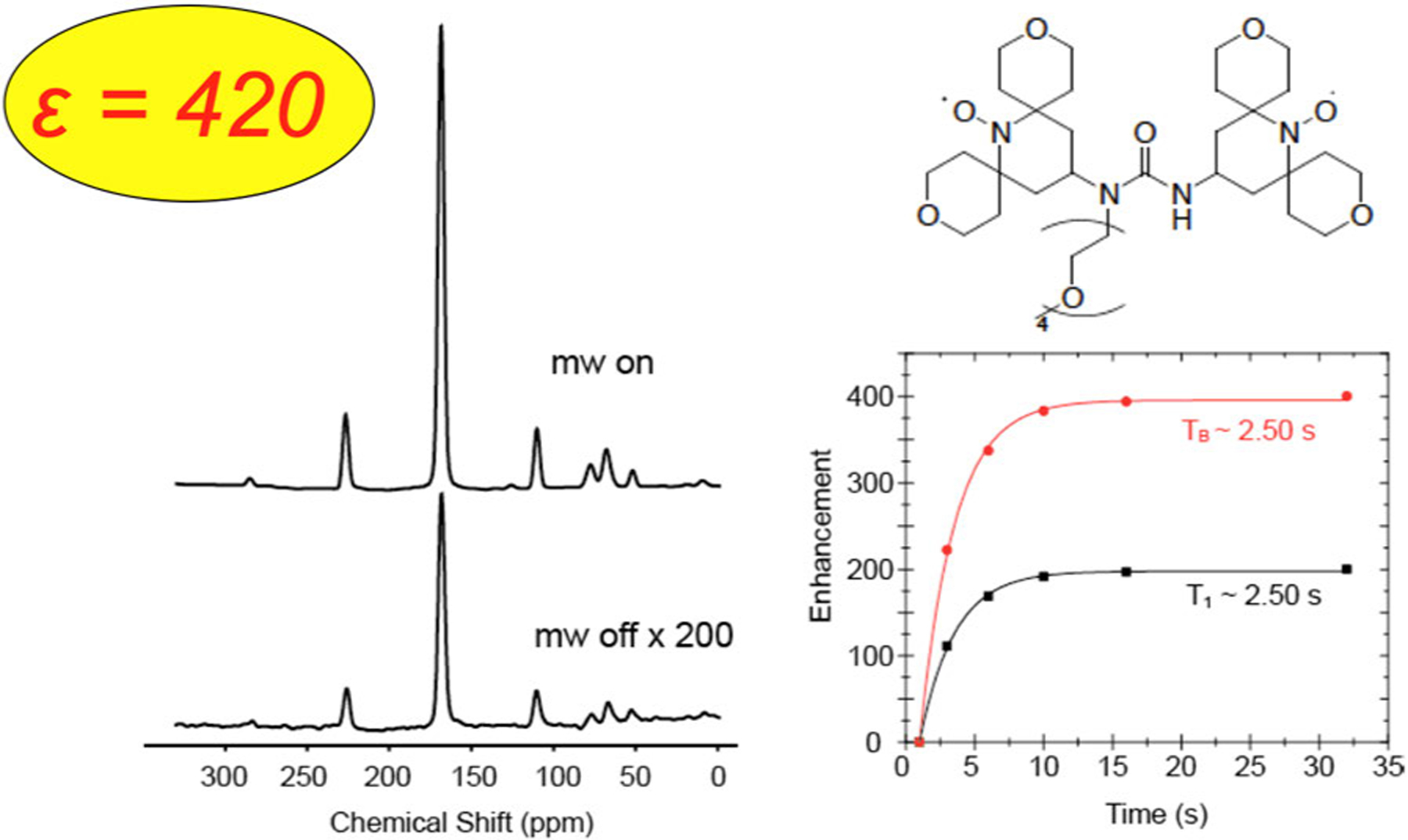
^13^C NMR spectrum (left) showing the DNP enhancement, a factor of 420 larger signal, produced by irradiation of the electron spin system of the biradical and the buildup curves of the signal (right). See also [19].

Solid-state nuclear magnetic resonance (NMR) is a powerful method for studying the structure of materials and biological systems such as proteins. However, the sensitivity of these studies is limited by the small polarization (~0.02%) of the nuclear spin states even at liquid nitrogen temperature. In principle, dynamic nuclear polarization (DNP) NMR can increase the signal-to-noise ratio of these studies by a factor of up to ~660. In DNP, a high-frequency microwave source excites the electron–nuclear spin transitions which then transfer the electron polarization to the nuclear spin system. An example is shown in Fig. 2, where a ^13^C signal is enhanced by a factor of ~420, which means an experiment that would require ~480 years of signal averaging without DNP can be completed in a day.

DNP was proposed and demonstrated in the 1950s [20], [21]. However, research in the field of DNP NMR did not progress to the high magnetic fields (≥5 T) used by the NMR research community until decades later, when the availability of high-frequency gyrotrons made modern DNP NMR research possible [22]. Since then, the field of DNP NMR has greatly expanded with many tens of installations of DNP NMR systems worldwide, as described in review articles [23], [24], [25], [26]. DNP NMR is frequently applied to study the structure of proteins, for example, fibrils associated with Alzheimer’s disease, to elucidate the structure of catalytic surfaces, and to imaging in clinical settings, for example of prostate tumors.

In DNP NMR, a source is required to excite the electron spins at their Larmor frequency = *egB*/2*m*, where *B* is the magnetic field, *e/m* is the electron charge/mass, and *g* is the electron magnetic moment = 2.002. The resolution of NMR spectrometers increases with magnetic field; the most common values are shown in Table I.

**Fig. 3.**
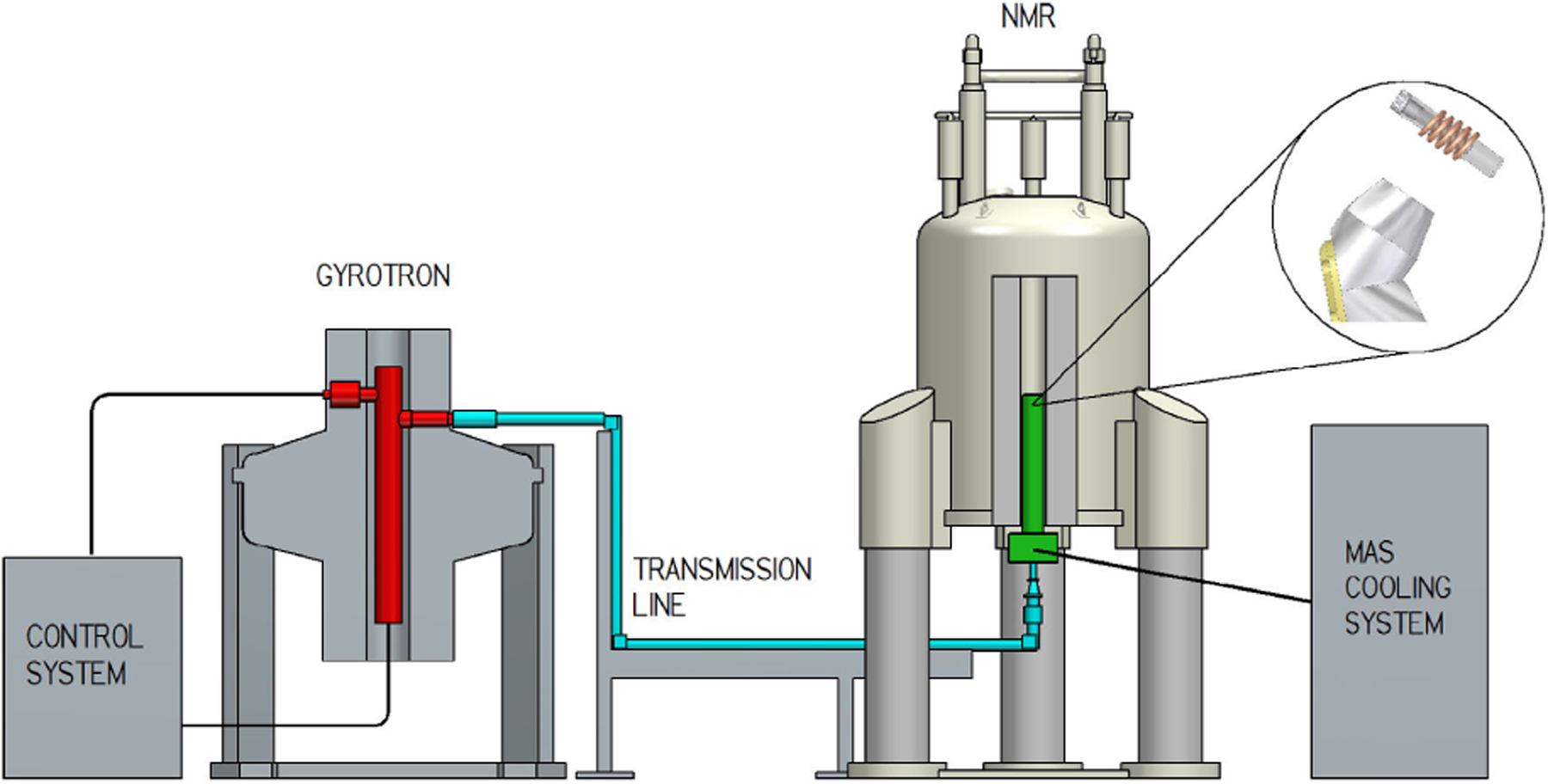
Schematic of a gyrotron-based DNP NMR system (reproduced with permission from [23]).

Fig. 3 shows the configuration of a commercial gyrotron-based DNP NMR spectrometer. The complete system requires more than just source power. The power must be coupled from the source and matched into a low loss transmission line, typically either a corrugated waveguide [27], [28] or a quasi-optical mirror line [29]. Coupling of the power into the sample holder is also a challenge since better coupling reduces the power requirements [30], [31].

As indicated in Table I, the required frequency for electron spin excitation falls into the sub-THz band. DNP NMR experiments acquire data in a continuous mode over a period of many days or even weeks. The THz source must produce stable output power meeting the minimum specifications listed in Table II.

Sources for DNP NMR have provided a significant challenge but also a major opportunity to the THz community. The most promising source has been the gyrotron oscillator and, to now, gyrotron oscillators meeting all of the requirements for application in DNP have been demonstrated up to a frequency of 593-GHz (900 MHz) DNP NMR spectrometer [32]. Research is already underway to provide gyrotron sources at even higher frequencies, up to 790 GHz [33], [34]. The required magnetic field for operation of a gyrotron is very close to the field of the matching NMR magnet. For very high-frequency gyrotrons, the cost of the magnet can be reduced by operating the gyrotron at the second harmonic of the electron cyclotron (EC) frequency.

**Fig. 4.**
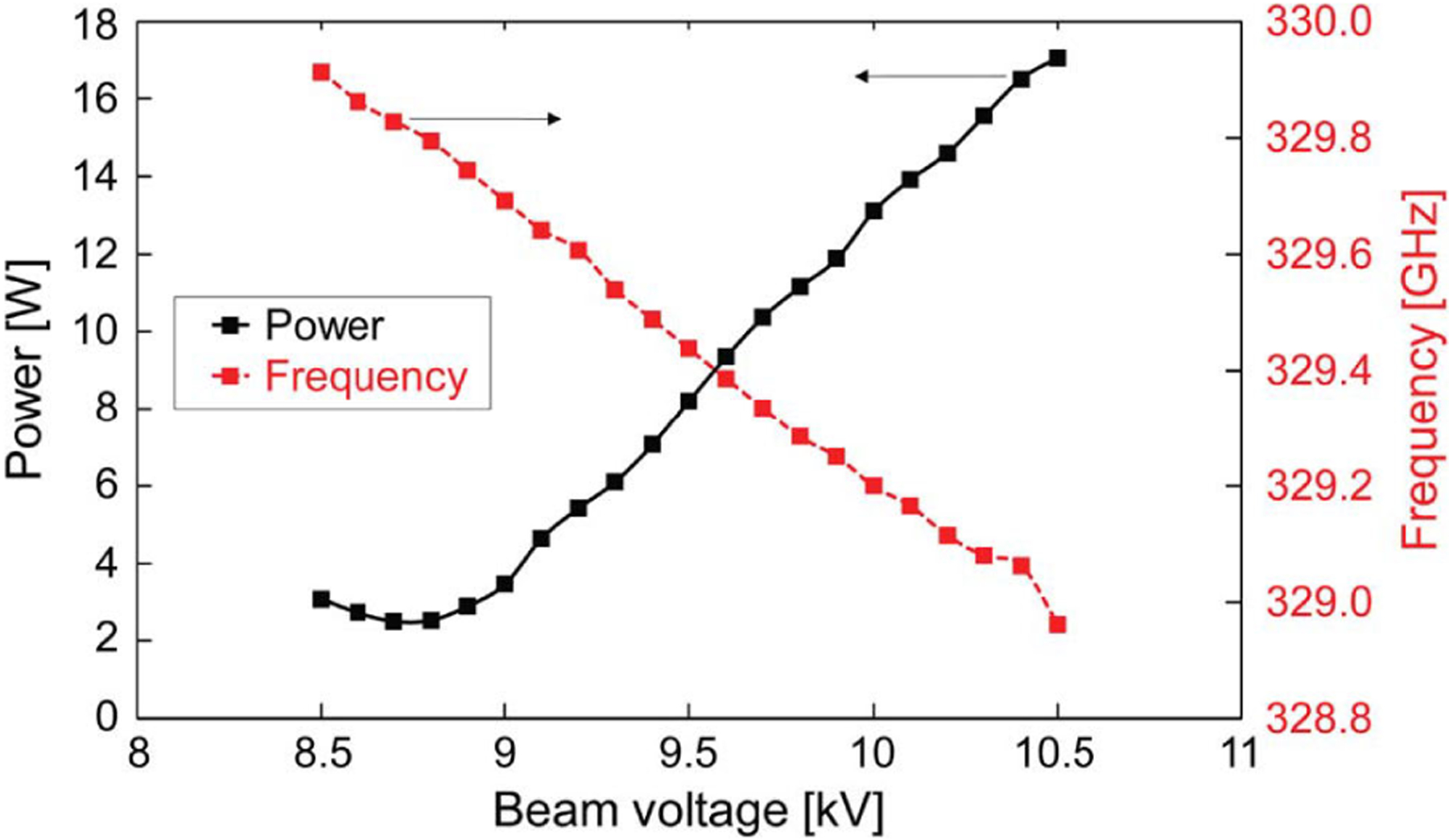
Tuning of a 330-GHz second harmonic gyrotron [40].

In addition to the requirements of Table II, the source must meet other requirements. If an NMR spectrometer is set for operation and lacks a sweep coil, the required THz frequency will be predetermined to within a few tens of MHz. The THz source may meet this requirement either by a very precise fabrication of the source or by building a source tunable in frequency. Frequency tunability of a THz source for DNP/NMR is thus highly desirable. Tuning also allows matching to specific resonances that may vary with the NMR sample or its mono- or bi-radical polarizing agents [35]. The gyrotron oscillator has limited tuning range since it has a very high quality factor cavity but may be tuned by one or even several GHz using a series of axial modes of the resonator. Hybridization of the axial modes can provide continuous tuning. An example of tuning a gyrotron in frequency by variation of the cathode voltage is shown in Fig. 4. Another approach that has shown promise is pulsing the cathode high voltage on the microsecond scale. The gyrotron frequency is swept by the changing cathode voltage in concert with the NMR frequency, allowing excitation of the full resonance spectrum in the time domain [36].

Sources other than the CW gyrotron oscillator are under intensive development for DNP NMR applications as well as for other applications such as radar [37]. An extended interaction oscillator (EIO) has been developed at 263 GHz with continuous output power in the 2–15 W range tunable over several GHz [38] and has now been used successfully for DNP NMR experiments [39]. The ability to site the EIO close to the NMR magnet offers an advantage in reducing the system footprint and in shortening the transmission line.

The challenge for the future will be the development of compact sources at higher frequencies, 395–790 GHz and above, needed for the highest field DNP NMR spectrometers. Significant DNP signal enhancements have been demonstrated with a power level as low as 150–250 mW from a solid-state source at 250–263 GHz [41], [42]. This may encourage the development of higher frequency sources even if power levels achieved are below the desired level of ~5 W.

Pulse sequences are useful for manipulating and detecting nuclear spins in DNP NMR research and electron spins in electron paramagnetic resonance (EPR). Both techniques shed light on molecular structure. To use pulse sequences, it is very advantageous to have amplifiers since they provide amplitude, phase, and frequency control of the microwave radiation. This is particularly important for pulsed experiments [43], [44], which have more favorable scaling for DNP enhancements at higher magnetic fields. Gyrotron amplifiers have been demonstrated at frequencies as high as 250 GHz [45] but have not yet been applied at the highest magnetic fields. A promising result was obtained with the amplifier version of the EIO, which has reached a pulsed power level of 72 W with a 1-GHz bandwidth at 263 GHz [46].

Gyrotron sources have also enabled new frontiers in EPR research at high magnetic field [47], [48]. Because the gyrotron can be tuned to produce high power at a sequence of frequencies, it allows greater flexibility in EPR experiments [49]. A pulsed EPR spectrometer at 240 GHz was developed using a free electron laser (FEL) as the source [50]. To reduce the length of the FEL pulses, a pulse slicer was employed using a laser-driven semiconductor switch to create 20-ns pulses for application in the spectrometer. An FEL is capable of reaching frequencies throughout the THz range, allowing the possibility of pulsed EPR at the highest possible magnetic fields in the future.

Advances in moderate power (watts to tens of watts) sources in the millimeter-wave (mm-wave)–THz range (200–800 GHz) have enabled major advances in magnetic resonance spectroscopy. Future developments, such as wide bandwidth amplifiers and more compact sources, will provide new, exciting possibilities for advances in this field.

### C. Wireless Communication: 5G and Beyond^3^

#### 1. Introduction

When envisioning an information world with fully realized next-generation communication networks, Hollywood-like applications such as “actionable” artificial/virtual reality and holographic communication immediately come to mind, but soon afterward the realization hits that ultrahigh tens of Tbit/s system data rates will be ultimately required. And while a captivating promise of 6G is to merge the human, physical, and digital world, that is the Internet of Things (IoT), there is the imposing need to make a jump by a factor of ten in connectivity density to ten million devices connected simultaneously per square kilometer.

The commercial realization of these exciting next-generation features depends on the deployment of high-density small cells for high capacity, a requirement especially crucial for operation in urban environments. A primary challenge facing these high data rate capillary-like network architectures is addressing the backhaul leg from base stations to core networks. Fiber is an option, but it is not present everywhere, expensive to deploy, and requires the permission of local authorities that is not always guaranteed. An attractive solution for capillary backhaul is to use wireless RF transmission, specifically operation in the mm-wave frequency bands (e.g., Q-band, E-band, and W-band) where wide accessible bandwidth crucial for multigigabit per second data rate transmission is accessible. However, the high signal attenuation of mm-wave radiation in rain or high humidity conditions results in high transmission power requirements at a level of tens of watts.

While the full potential of 5G can be achieved through operation in mm-wave bands in the 24.25–52.6-GHz frequency range, the next generation of 6G mobile connectivity likely requires the move to frequencies above 100 GHz, and possibly into the true terahertz, to achieve the 1-Tb/s peak data rates required for holograms and enhanced virtual reality experiences [51].

Another exciting opportunity brought about by the incorporation of mm-wave and THz bands in future 6G networks is to integrate a sensing function into the communication system. Known as integrated sensing and communication (ISAC), it offers the benefits of including both cellular as a sensor and sensing-assisted communication [52].

While it is quite challenging to provide a clear vision for 6G, it is fairly certain that it will continue the trend of using higher carrier frequencies beyond the mm-waves through THz bands and up to visible light, to provide high-capacity point-to-point communication with an aim to achieve spectral efficiency 5× greater than 5G. The lower sub-THz band, with frequencies in the 100–200-GHz range, seems like a good candidate for such applications. Researchers and engineers are already conducting studies and developing devices and systems in D-band (110–170 GHz) and G-band (140–220 GHz). True THz communication beyond 300 GHz may come, but it may only be in time for 7G. The biggest challenge is the achievable link distance, as transceiver available output power and sensitivity are expected to be low for the foreseeable future [53].

#### 2. Technology Demonstration Projects

A number of projects have been conducted, or underway, around the world aimed at demonstrating the capability of next-generation networks, as well as developing the key technology components. One of the first was the European Union’s H2020 TWEETHER (traveling wave tube (TWT) based W-band wireless networks with high data rate, distribution, spectrum, and energy efficiency) project [54]. TWEETHER aims at demonstrating a W-band (92–95 GHz) point-to-multipoint system for backhaul with high capacity access. A related project is ULTRAWAVE (ultracapacity wireless layer beyond 100 GHz based on mm-wave travelling wave tubes), which is aimed at developing a high-capacity backhaul through 5G cell densification by exploiting frequency bands above 100 GHz [55]. The vision for this program is the creation of an ultrahigh capacity layer providing more than 100 Gb/s per square kilometer in point to multipoint at D-band (141–174.8 GHz) fed by novel G-band (300 GHz) point-to-point high-capacity links. The ULTRAWAVE layer will enable backhaul to hundreds of small and pico-cells, no matter the density, opening scenarios for new network paradigms aiming at a full 5G implementation. Finally, a recent program, called THoR (terahertz endto-end wireless systems supporting ultrahigh data rate applications) [56], aims to provide technical solutions for the data networks beyond 5G based on 300-GHz RF wireless links.

For high data rate wireless communication, the use of carrier properties including frequency, phase, and polarization has been adopted for modulation in the transmission system. Recently, orbital angular momentum (OAM) was studied for future mobile communication. OAM employs a spatially modulated radiation beam with a helical wavefront and phase singularity at the center. It consists essentially of an infinite number of orthogonal modes, quantified by the so-called topological charge relating to the number of twists of the wavefront in one wavelength, which propagate independently. As a result, the capability of the information carried at one frequency is significantly increased [57]. During the Beijing Winter Olympic Games in January and February 2022, researchers from Tsinghua University, Beijing, China, successfully conducted an OAM demonstration experiment at W-band. A data rate relay transmission of up to 1 Tb/s was achieved at a maximum transmission distance of 1 km. The experimental wireless communication line can support more than 10 000 live high-definition video streams simultaneously [58]. This experiment verifies the feasibility of point-to-point data transmission using OAM technology and supplies the foundation of experimental data for use in future 6G mobile communications. Recently, by exploiting the merits of high-power capability and customizable radiation frequency and high-order topological charges over a broad bandwidth, intense vortex Smith–Purcell coherent radiation has been demonstrated with steerable OAM using free electrons and helical interaction structure, thus paving the way for high-power, tunable OAM sources based on vacuum electronics [59].

#### 3. TWT Development for Next-Generation Communication

While solid-state RF amplifiers have a clear role to play in next-generation communication network architectures, and photonics, too, TWTs have an advantage when it comes to the legs of the distribution where high power is required. This is especially true for systems operating in the millimeter and THz frequency bands where TWTs can deliver power from the hundreds of watts at mm-wave to tens of watts to watts in THz. TWTs are also more efficient than the other source alternatives. Besides designing TWTs for the requisite power requirements for wireless terrestrial communications, considerable effort has recently been made to increase TWT bandwidth, efficiency, linearity, and reliability, and to decrease the noise figure, size, and weight of the packaged amplifier (including HV power supply), as well as its cost, all of which are obvious concerns to network equipment suppliers and service providers.

**Fig. 5.**
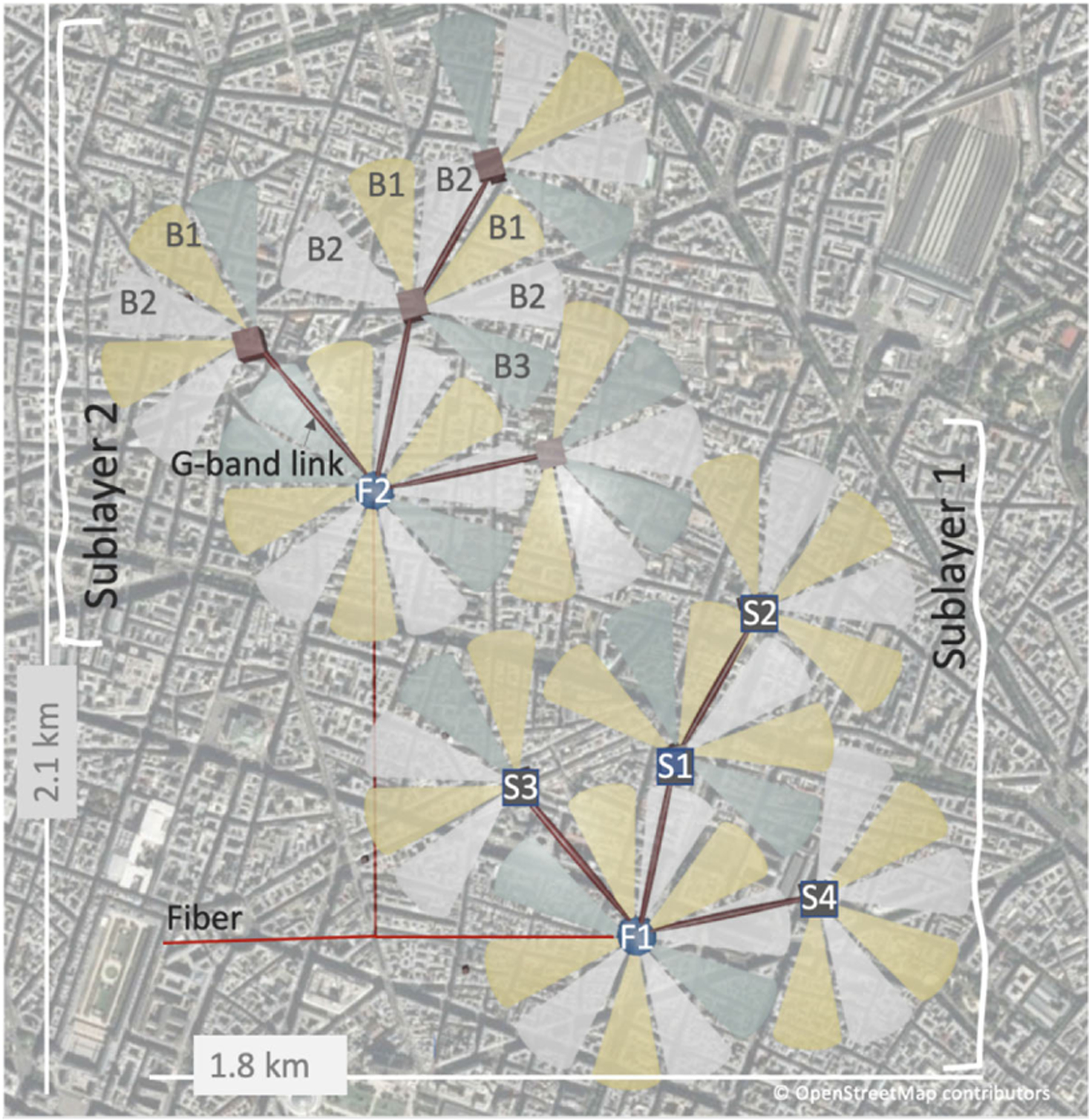
Deployment of wireless transport layer in urban scenario (top view). F1 and F2 are fiber access points; S1, S2, S3, and S4 are D-band clusters; and B1, B2, and B3 D-band are subbands. G-band links in red [60].

TWTs at Q-band (37–41 GHz), E-band (81–86 GHz), W-band (92–95 GHz), and D-band (141.5–178.4 GHz) can be used for point-to-multipoint distribution as a wireless distribution layer (WDL), while higher frequencies, e.g., G-band, can be used for point-to-point wireless communication or constructing the wireless transmission layer (WTL) placed on top of the WDL in cooperation with the fiber substrate. The main features of the combination of the WDL with WTL, powered by TWTs, are easy installation, total cost of ownership competitive to fiber, high signal-to-noise ratio and spectral efficiency, and low latency. The WTL will be at the same time complementary and an alternative to fiber [60]. A notional implementation of a multilayer network for an urban environment is shown in Fig. 5.

For high data rate communication, L3 (now Stellant Systems, Torrance, CA, USA), THALES, Vélizy-Villacoublay, France, and BVERI, Beijing, China, have reported TWTs or microwave power module (MPM) amplifiers with operating frequencies greater than 70 GHz, and up to 340 GHz [61], [62], [63], [64], [65]. These devices operate CW providing high power for longer distance transmission; wideband with high linearity for high data rate, high-quality communication, and high efficiency for base station use with reduced energy consumption. Essentially all of the TWTs used at these mm-wave/THz frequencies employ an all-metallic circuit structure, such as folded waveguide or double corrugated waveguide, due to their high-power handling capability and promise of mass manufacturing.

#### 4. Conclusion

Millimeter wave and THz communication will clearly be part of 6G; however, high transceiver power is required; otherwise, it is only expected to be deployed for specific uses, such as indoor connectivity, interdatacenter communications, and unmanned aerial vehicle (UAV)/satellite communications. Vacuum power devices based on the interaction of free electrons with a vacuum electromagnetic field can deliver much higher power than solid-state amplifiers, especially in the projected mm-wave and terahertz communication bands. The 2-D structure features of the all-metal, periodic TWT folded waveguide and vane-loaded waveguide circuits used at these high frequencies coupled with their small dimensions of tens of microns make manufacturing compatible with modern microfabrication processes, including nano-computer numerical control (CNC), ultra violet lithographie galvanoformung and abformung (UV-LIGA), deep reactive ion etching (DRIE), and 3-D printing with high precision of micrometers and high uniformity. However, surface roughness, density, and oxygen content of material made through microelectromechanical systems (MEMS) and additive manufacturing need attention for compatibility with use in vacuum devices. In addition, improvement of TWT bandwidth, linear power, and noise properties with regard to modern digital communication requirements must be addressed. Similarly, optimization of HV operating voltage, miniaturization, and efficiency of integrated power amplifier modules for base station use is important for system service and maintenance.

MM-wave and THz TWTs can play a critical role in powering future wireless communication networks; improvement remains on specification definitization, and on device reliability, lifetime, and cost for full-scale system adoption.

### D. Microwave and Millimeter-Wave Vacuum Electronics for Space Applications^4^

#### 1. Introduction

In recent years, the European Space Agency (ESA), Noordwijk, The Netherlands, together with other national space agencies has funded a number of research and development projects in the field of space TWTs. These developments cover a broad range of applications, spanning from telecommunications and Earth observation to deep space communication. Together, these developments push the envelope of what is currently possible with vacuum tube technology and secure Europe’s capability to deploy the state-of-the-art space missions for the years to come.

#### 2. Telecom Applications

A major field of interest for ESA is to provide fast, stable, and secure telecommunications to the citizens, businesses, and public entities of its member states. For space telecommunication links, Ku-band and Ka-band are the predominant bands. Therefore, the focus of vacuum tube development has been on high-power TWTs and very compact dual mini-TWTs, both in Ku- and Ka-band.

For high-power telecommunication, Ku- and Ka-space TWTs providing over 250 W with efficiencies of over 64% have been developed. Both conduction and radiation cooled versions have been realized. As this development has been reported extensively in the literature, the interested reader is directed to consult [66] and [67].

**Fig. 6.**
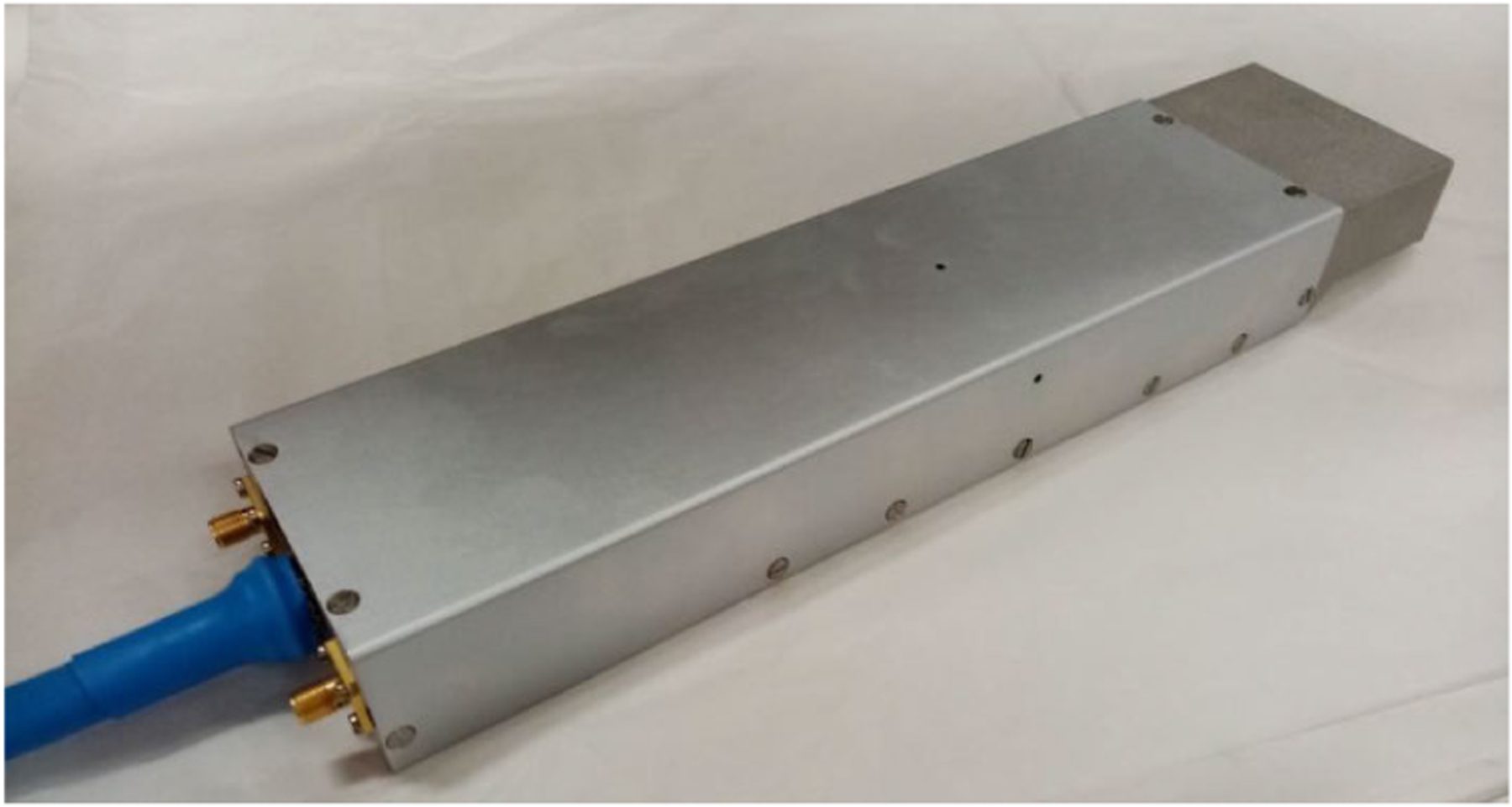
Engineering model of Ku-band dual mini-TWT. The mass of the dual-TWT (without cable) is under a kg.

To progress in a new generation of efficient, highly compact tubes, Thales has developed the dual-TWT. An engineering model of which is shown in Fig. 6. It consists of two miniaturized TWTs that are mounted together on the same baseplate and fed by a single electronic power conditioner (EPC) and high-voltage cable. Through the use of this architecture, a product with a very small form factor can be achieved while staying close to the good performance of the established Ku and Ka-band TWTs. In Ku-band for example, saturated output powers of >70 W are reached with an efficiency of >60% while keeping the mass of the dual-TWT (without cable) below 1 kg.

Achieving this good performance is possible not only due to the design effort but also due to the employment of additive manufacturing, which is used to fabricate the baseplate and output waveguide. Through successfully reducing the size of the TWTs, the dual-TWT is compatible with the stringent lattice spacing requirements of active antennas and at the same time outperforming solid-state power amplifiers (SSPAs) in terms of output power, efficiency, and linearity, paired with the excellent reliability of TWTs. On top of the excellent performance, the dual-TWT will have the capability of flexible output power. The first successful development was completed for Ku-band in 2022 and the next step will be to develop a dual-TWT for Ka-band [68].

A comparable U.S. product for satcom Ka-band with 60 W of saturated output power is also available from Stellant Systems [69].

##### a. High Frequency Telecom

With telecom operators moving to higher frequencies for the feeder links of future satellite communication systems, ESA is also supporting the development of Q- and W-band TWTs for space. The advantage of going to higher frequency bands lies in the larger instantaneous bandwidths, which allow for higher data throughput. With their capability of covering large bandwidths and with SSPAs facing difficulties to achieve high-power levels at those frequencies, TWTs are currently the technology of choice for power amplification at frequencies above Ka-band.

Thales has successfully developed an 80-W Q-band (37.5–42.5 GHz) TWT with a gain of 80 dB and a saturated efficiency of around 50% [70]. The next step for reaching an even higher instantaneous bandwidth and thus higher transmission speeds will be to use W-band (71–76 and 81–86 GHz) [71].

##### b. Deep Space Communication

While the research in the field of telecommunications TWTs is mainly focused on boosting the capabilities and the competitiveness of the European vacuum tube industry, ESA is also funding TWT research oriented toward its own needs in the fields of space exploration and Earth observation.

For the communication links of future deep-space exploration missions, ESA plans to make use of the Space Research Service (SRS) Ka-band downlink, because of its large bandwidth (31.8–32.3 GHz) and the fact that, as of 2022, it is not very crowded yet. Since these are projected to be equipped with a high-gain antenna and a single RF source, TWTs are the ideal candidates for the high-power amplifier (HPA).

One of the first missions that will make use of this frequency band with its telecommand, tracking, and control transponder will be the ESA/NASA EnVision mission to Venus in 2031 [72].

With currently no TWT in Europe being able to fulfill the power requirements of that mission, predevelopments have started. The requirements are 500 MHz of instantaneous bandwidth, more than 120 W of saturated output power, an efficiency of better than 50% and a lifetime of at least seven years in the operational environment. To be compatible with a currently existing heritage EPC, the cathode voltage of the TWTs is limited to 10 kV.

To start the predevelopments, two parallel contracts have been initiated in 2020, one with Thales AVS, Vélizy-Villacoublay, France, and one with Leonardo Airborne and Space Systems, Palermo, Italy. Excellent results have been achieved by both contractors, and the target technology readiness level (TRL) reached by 2022 is TRL 4.

#### 3. Earth Observation

ESA is a world leader in Earth observation and remains dedicated to developing cutting-edge spaceborne technology to further understand the planet, improve daily lives, and support effective policy-making for a more sustainable future that also benefits businesses and the economy as a whole.

For the observation of Earth, most of these satellites make use of active microwave sensors. The choice of frequency is dictated by the physics of the relevant scattering mechanism.

Three missions, at different stages of development, are described in the following. All of them have instruments using klystron tubes.

##### a. WIVERN

Wind Velocity Radar Nephoscope (WIVERN) is a candidate mission that would have a spaceborne dual-polarization, 94-GHz Doppler radar, conically scanning at an off-zenith angle of 4°. It measures the in-cloud line-of-sight winds over an 800-km-wide ground track using the returns from cloud and precipitation targets, satisfying the World Meteorological Organization (WMO) requirements for numerical weather prediction models of horizontal winds with 2-m/s accuracy, ~50-km horizontal and 1-km vertical resolutions, and daily sampling poleward of 50° [73].

WIVERN would use two of the same 94-GHz transmitter tubes that CloudSat [74] has operated, well beyond expectation in lifespan, since the 2006 launch, with 3.3-*μ*s pulsewidth and a similar pulse repetition frequency (4 kHz). The tube is an extended interaction klystron (EIK) capable to deliver 2-kW peak RF output power, manufactured by Communications & Power Industries (CPI) Canada, Georgetown, ON, Canada.

##### b. SWOT

Surface water and ocean topography (SWOT) is a mission resulting from a joint effort of NASA and the Centre National d’Études Spatiales (CNES) (French Space Agency), Toulouse, France, with contributions from the Canadian Space Agency (CSA), QC, Canada, and the U.K. Space Agency (UKSA), Swindon, U.K.

**Fig. 7.**
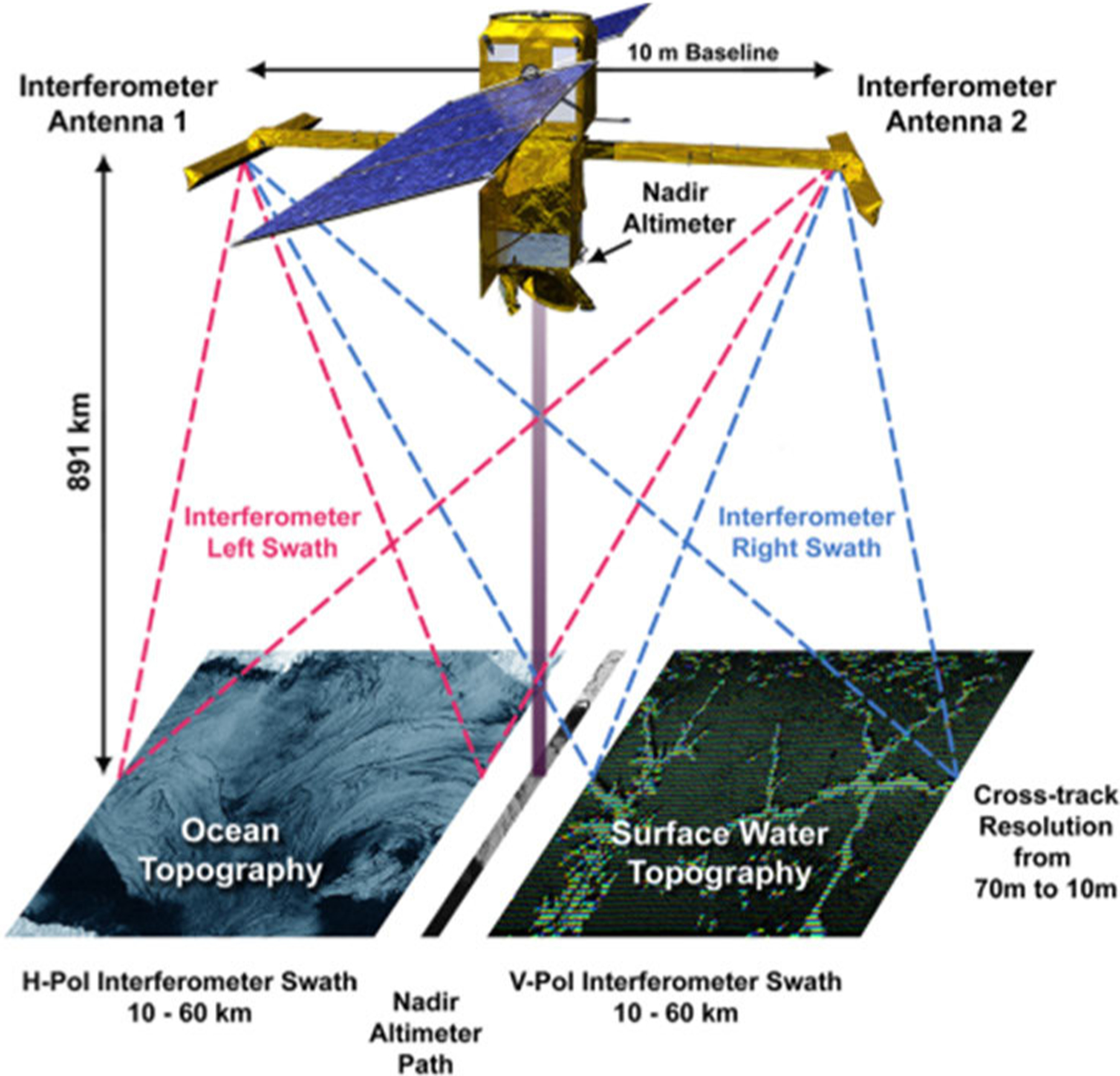
SWOT dual-swath Ka-band radar interferometry system.

Soon to be launched, SWOT will collect data on ocean water levels to study currents and eddies up to five times smaller than have been previously detectable. It will also gather detailed information on freshwater lakes and rivers. Water height is measured by a wide-swath interferometric synthetic aperture radar (SAR) operating in Ka-band (see Fig. 7) [75]. The instrument operates at 35.75 GHz with an instantaneous transmission bandwidth of 200 MHz, 4.5-*μ*s pulsewidth, and 4.4-kHz pulse repetition frequency.

The HPA consists of a 1.5-kW RF power amplifier (EIK) supplied by the CSA and developed by CPI Canada and a 15-kV high-voltage power supply (HVPS), developed by the Jet Propulsion Laboratory, Pasadena, CA, USA.

##### c. MetOp SG

MetOp-Second Generation (MetOp-SG) is EUMETSAT’s next generation of polar-orbiting satellites and a follow-up to earlier successful missions. The mission is composed of two series of spacecraft, Metop-SG A and B, flying on the same mid-morning orbit as the current Metop satellites in the range of 823–848 km with three satellites of each type A and B.

A key instrument of Sat-B is the wind scatterometer (SCA). The primary scientific product of the SCA is wind vector data, derived from radar cross-section measurements of the ocean surface by using a geophysical model function (GMF). SCA will cover 99% of the Earth’s surface within 48 h, using a swath width of 625 km on each side of the satellite track and a resolution better than 25 km^2^ [76].

SCA is equipped with six array antennas, each of them consisting of four panels fed by a network of waveguides. The antennas are accommodated on three antenna assemblies. With respect to the spacecraft flight direction, these three antenna assemblies are oriented to broadside (mid beam) and ±45° from broadside (fore and aft beams, respectively) so that they allow for sequential observations of the backscattering coefficient of each point of interest on the left and right swaths from three directions.

The RF instrument operates at 5.355 GHz and requires 2.2 kW peak RF output power with an unusual 1.15-ms pulsewidth and 32-Hz pulse repetition rate.

The HPA consists of an EPC developed by Airbus DS Electronics Department, Friedrichshafen, Germany, and a four-cavity klystron developed by CPI Canada operating at 11 kV with an overall efficiency of 40% using an undepressed collector. The tube is capable to deliver 2.2 kW peak RF output power with 32 dB gain [77]. A tube with very similar characteristics has been developed in Europe by Leonardo Airborne and Space Systems [78].

### E. Microwave Technology for Fusion Applications^5^

#### 1. Introduction

**Fig. 8.**
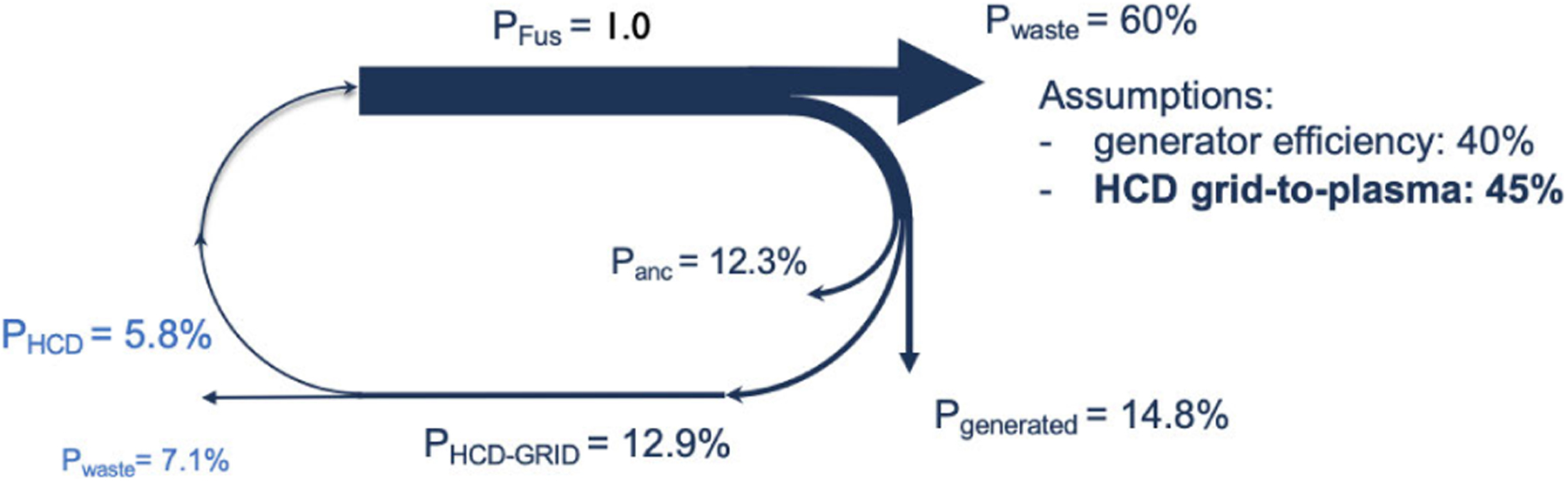
Example of a fusion plant Sankey diagram assuming a reactor generating ~1.5 GW of fusion power (*P*_FUS_), roughly <40% of that power would be used to generate the electricity. The majority of that generated power is recycled to maintain the plant ancillary systems (*P*_anc_) and the HCD system (*P*_HCD-GRID_), while <15% as net electrical power to the grid.

This work in magnetic confinement fusion is facing a change toward the next-generation devices that aim at a net energy gain to the electrical grid. Current devices have approached a net energy balance as viewed by the plasma but have eluded the limit of *Q* = 1 (energy generated/energy injected into the plasma) with the best results achieved in the Joint European Tokamak (JET) [79]. The larger device International Thermonuclear Experimental Reactor (ITER) [80] aims at a tenfold increase (*Q* = 10), but the device, though expected to validate some key concepts needed for commercial viability, is experimental and does not attempt to generate electricity. A fusion generating plant would require *Q* > 10, to compensate for the large recirculating power to run the device and the low efficiencies in the steam turbine (typically 30%–40%). A typical Sankey diagram normalized to the fusion burn product is illustrated in Fig. 8.

For a tokamak device generating 1.5 GW fusion power, roughly one-third of the fusion burn is used to generate the electricity to maintain the heating and current drive (HCD) power of ~150 MW (injected) to achieve the required high plasma temperatures (~1.5 × 10^8^ °C) and sustain an internal current for confinement. The more efficient the HCD system, the less the parasitic load on the plant with more energy being supplied to the grid.

Future devices such as the various demonstration reactors (DEMO) [81], [82], [83] planned by the ITER partners and the U.K.’s Spherical Tokamak for Energy Production (STEP) [84] are assessing the various methodologies for plasma HCD, which include momentum transfer using neutral particle accelerators (or neutral beams, NBs), radio waves with frequencies matching the ion cyclotron (IC) frequency (10–100 MHz), or microwaves matching the electron cyclotron (EC) frequency (70–300 GHz). The choice depends largely on the machine parameters and its requirements for either plasma heating and/or current drive applications, the net efficiency of taking power from the grid to converting it for the given HCD mechanism, and the engineering challenges of systems in the proximity of the burning plasma.

#### 2. Electron Cyclotron Heating and Current Drive

**Fig. 9.**
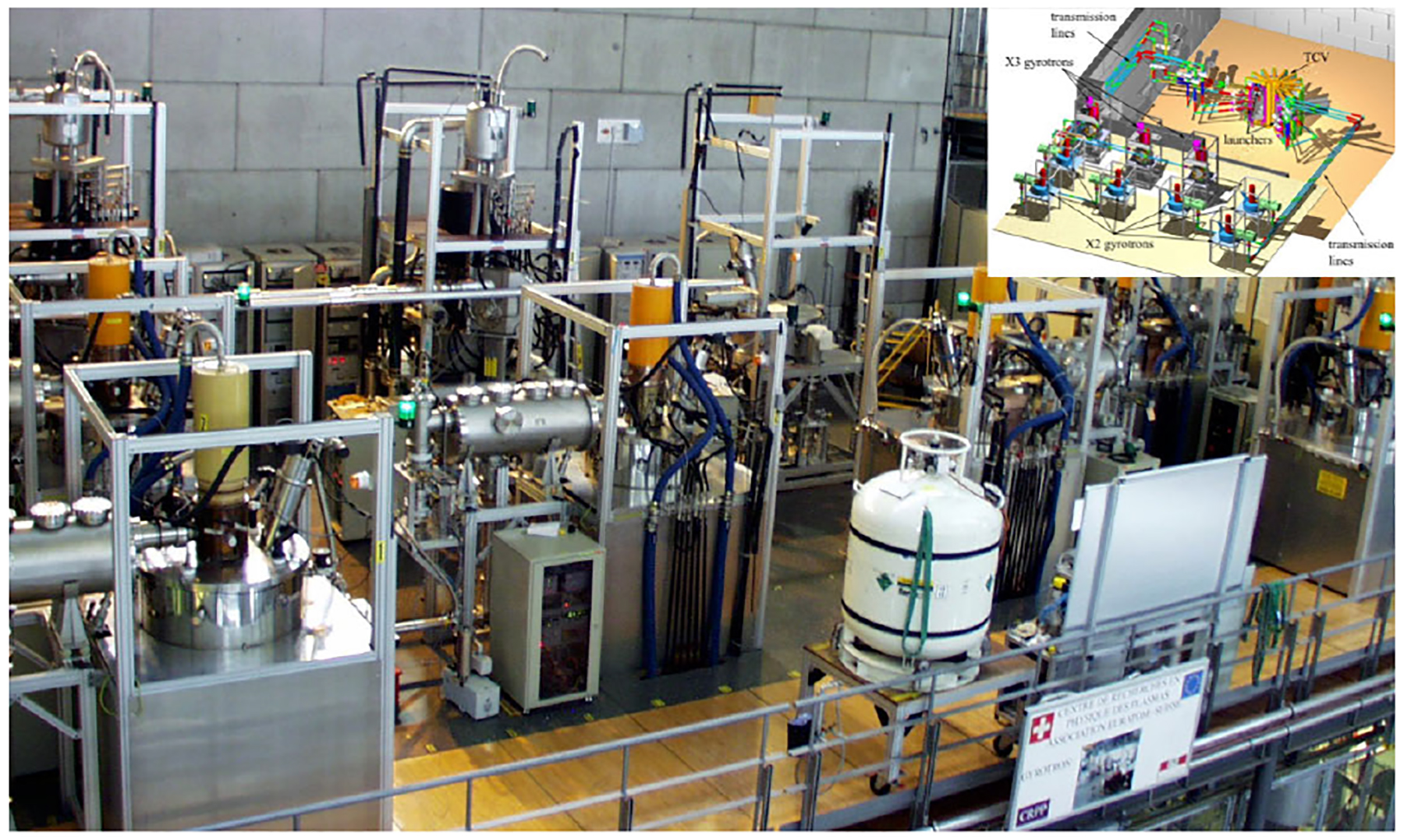
Image of the TCV’s 4.5-MW EC HCD system [87], [88], which is located ~30 m from the tokamak center (inside concrete wall). A reference of the plant layout is illustrated in the top right inset.

The EC HCD is the least challenging engineering system as there are a minimum number of components in the proximity of the burning plasma. The microwave sources (or gyrotrons) and their paired HVPSs need be several tens, or even hundreds, of meters distance from the fusion device, as its magnetic field required to achieve plasma confinement impacts the gyrotron operation if these are placed in close proximity. The microwave power is transmitted to the fusion device via evacuated waveguide [85] or quasi-optical [86] transmission systems. The 4.5-MW EC HCD system [87], [88] on the Tokamak â Configuration Variable (TCV) is an example of an operating plant, as shown in Fig. 9.

Inside the tokamak vacuum chamber, the microwaves can be transmitted via a Gaussian beam, propagating as under the paraxial approximation, with quasi-optical mirrors directing the beams via a labyrinth and through the plasma facing blanket shield module (used for dampening the 14 MeV neutrons). Such a configuration maintains a relatively simple mechanical system in-vessel that has limited thermal and nuclear loads, and achieving increased longevity as compared to the other HCD concepts. The transmission efficiency from source output to coupled power to the plasma can achieve >90% power transmission efficiency.

As the microwaves “heat” the plasma by matching the local cyclotron frequency of the electrons, which have small Larmor radii (<1 mm), the deposition can occur over a relatively small region. Using quasi-optical mirrors, the beams can also be focused to small diameters offering a surgical tool [89] that can heat locally and drive current for tailoring the plasma parameters and/or stabilizing plasma instabilities [90].

There are limitations in the capabilities of an EC HCD system, for example, the ion heating required for fusion is only achieved via electron–ion collisions. The Lawson criteria [91] describes the dependence between ion temperature, ion density, and the energy confinement time to achieve reactor relevant conditions. EC HCD is at a disadvantage relative to IC or NB HCD systems, as these systems contribute a portion of their power to direct ion heating. This handicap can be overcome with increased plasma densities that increase the energy transfer between the electrons and the ions.

EC HCD will play a primary role in future commercial fusion plants as its functional capabilities and more relaxed engineering challenges exceed that of other HCD mechanisms. This includes the overall “plug-to-plasma” efficiency, which is the ratio of the delivered microwave power to the total power taken from the grid to generate and transmit that power to the plasma. Today’s technologies are limited to <40% “plug-to-plasma” efficiencies, however, to render an EC HCD system compatible with fusion commercialization, the efficiency should be ≥45%. As the main limiting factor is the source efficiency (~45%–50%), future research and development should focus on improving the gyrotron efficiency along with increased reliability and availability.

##### a. Gyrotrons

The gyrotron device [92], [93] has been the primary source concept for EC HCD applications to date, providing unit powers up to ~1 MW in the range of 30–170 GHz for pulse lengths up to 1000 s [94], [95], [96]. Several cylindrical cavity designs have yielded multifrequency sources with similar output performance [97], [98], [99] and higher unit powers ~1.5 MW, albeit at shorter pulse lengths [100]. Accessing higher power sources is limited by the thermal loading in the cavity, which requires larger diameter cavities to keep the thermal loading <~20 MW/m^2^. However, a larger diameter cavity and simultaneously higher EC frequencies (140–300 GHz) require higher order TE_*m,n*_ cavity modes. But with ever increasing *m, n* mode number, the competition between neighboring modes increases. This can be overcome by placing a co-axial rod along the axis of the cavity to limit the mode competition. It allows for significantly larger so-called “coaxial” cavities, paving the way for higher power sources of ≥2.0 MW unit powers [101]. In addition, the co-axial insert can be used to achieve step-tunable frequency sources (Δ*f* ~ 1 GHz) [99].

Advanced operation schemes have also been achieved, using either phase locking [102] or active predictive mode jumping assessments, allowing the gyrotron to operate closer to its optimal performance parameters [103].

#### 3. Future Technology Needs

The improved power and frequency control offers improved experimental capabilities for near term devices, where the “plug-to-plasma” efficiencies are not primary requirements. However, as commercial fusion reactors become feasible, the key design driver will be toward increasing the electrical to microwave power efficiency, which minimizes the parasitic loads of the fusion plant, as illustrated in Fig. 8.

The development of single-staged depressed collector (SDC) [97], [98] has improved the source efficiency approaching ~50%. This is achieved by elevating the cavity potential (+30 kV) to maintain an electron beam acceleration voltage to ~80 kV, while the main power supply between the cathode and collector remains lower, 50 kV. The collector can be further subdivided into multiple bias voltages to maximize the energy recovery using the multistaged depressed collector (MDC) design [104]. The use of MDC gyrotrons offers a theoretical efficiency approaching ~65%, which would improve the EC HCD plant efficiency toward ~58%, significantly reducing the parasitic load on the fusion plant.

An additional gyrotron enhancement achieves direct coupling to HE_11_ waveguide [105], offering further improvement to the overall efficiency by removing the matching optics unit (MoU). The MoU is comprised of ≥2 quasi-optical mirrors to convert the gyrotron output beam into the ideal phase and amplitude to couple to the HE_11_ waveguide (assuming the use of evacuated waveguide for the transmission line). Removal of the MoU also reduces the gyrotron cost and simplifies the installation and maintenance procedures.

Further advancements in gyrotron operation reliability and longevity are needed. As a tokamak fusion plant will require between 100 and 150 MW of continuous auxiliary power, pulse lengths will be required that last years rather than 15 min in present source capabilities. Also needed is extension of the gyrotron life expectancy with increased cathode operating times exceeding 100 000 h, along with more component modularity to achieve rapid repair times.

Over the next 20 years, the required development of sources based on reliability, availability, maintainability, inspectability (RAMI) principles, with high reliability and availability, while simplifying the maintenance and repair down times is essential. The past 20 years have shown significant improvement in source development, but the next 20 years will require an equal, if not greater, level of industrialization improvements for fusion power to be realized.

## III. Conclusion

From new cancer treatments to progress on the realization of commercial fusion power, from high-speed, high-capacity next-generation communication networks to flexible high-throughput satellite communication and remote earth observation, and to high-resolution/high-speed magnetic resonance imaging for advanced material and bio-science investigations, RF vacuum electronics continues to play an integral role. A general recurring theme apparent in the application write-ups is a push to higher frequency, specifically from microwave to mm-wave, and beyond. As seen by the institutional affiliations of the authors of the application summaries, the pursuit in the use of vacuum electronics for improving the societal condition is truly international in scope.

As mentioned in Section I, while an attempt was made to cover a wide and diverse application landscape within print limitations, some areas had to unfortunately be omitted. For example, the areas of national security and high-energy physics, long-time legacy users of RF vacuum technology were not covered. Similarly, the active area of high power microwave (HPM) [106], which spans the technology boundary between pulsed power and conventional vacuum electronics, was also not discussed. HPM is a subset of the general topical area of directed energy. Other uses in this area where vacuum electronics can have a role are in wireless power beaming [107] and even possibly the more speculative areas of subterranean drilling for tapping geothermal energy [108] and rocket booster propulsion [109]. Finally, the field of vacuum nanoelectronics, which includes the pursuit of RF vacuum devices with nanometer scale structure features, remains of interest around the world [110], [111]. It is safe to say that the application of vacuum devices will continue in the future, driven by humanity’s need and imagination.

## Figures and Tables

**TABLE I:** DNP NMR Frequencies

B(T)	NMR Freq. (MHz)	Electron Freq. (GHz)
9.4	400	263
14.1	600	395
18.8	800	527
23.5	1000	658
28.2	1200	790

**TABLE II:** DNP NMR Source Requirements

Source Power	≥ 5W; 20 W desirable
Power Stability	± 1%
Frequency Stability	Drift of < 10 ppm

## Footnotes

1 Authors of this section are E. C. Snively, M. Shumail, C. Nantista, Z. Li,S. Tantawi, and B. W. Loo.

2 Authors of this section are R. J. Temkin and R.G. Griffin.

3 Author of this section is J. Feng.

4 Authors of this section are R. Dionisio, F. Mentgen and N. Ayllon.

5 Authors of this section are M. A. Henderson and T. P. Goodman.
